# RS1799817 in INSR associates with susceptibility to polycystic ovary syndrome

**DOI:** 10.2478/jomb-2019-0023

**Published:** 2020-01-23

**Authors:** Maha H. Daghestani

**Affiliations:** 1 King Saud University, College of Science, Department of Zoology, Riyadh, Saudi Arabia

**Keywords:** single nucleotide polymorphism, insulin receptor gene, polycystic ovary syndrome, reproductive hormones, lipid profile, profil lipida, reproduktivni hormoni, sindrom policističnih jajnika, GEN receptora insulina, jednonukleotidni polimorfizam

## Abstract

**Background:**

Insulin and its receptor (*INSR*) have been implicated in the etiology of the polycystic ovarian syndrome (PCOS). Here, we investigate the association between *INSR* rs1799817 polymorphism and PCOS in Saudi Arabian women.

**Methods:**

Study group included 126 PCOS women and 118 normo-ovulatory matched controls. The demographic data was recorded, and the plasma levels of glucose, lipids, leptin, E2, LH, FSH, T, SHBG, and insulin were determined. The genotypic and allele frequencies of rs1799817 were evaluated in both PCOS and control group. Polymerase chain reaction (PCR) was used to amplify Exon 17 of the *INSR* gene, and the amplified products were analyzed by direct sequencing. A single-nucleotide polymorphism (C to T) was found at locus 10923 (His1058) of rs1799817.

**Results:**

In the PCOS group, the mutant allele T occurs at a significantly higher frequency (0.306) compared to the control group (0.174) (p<0.001). It shows a dominant effect and elevates the relative risk of PCOS even in the heterozygotes (RR=2.82). After stratification of the participants by body mass index, the frequency of T allele was significantly higher in the lean patients with PCOS compared to the lean control. The obese PCOS also had a higher frequency than the obese control, but the difference was not statistically significant. Several parameter values were affected by the INSR genotype, particularly W/H ratio, lipid, insulin and glucose levels and insulin resistance in PCOS patients.

**Conclusions:**

The INSR gene polymorphism rs1799817 is a susceptibility locus associated with PCOS in Saudis and associated metabolic and hormonal changes, particularly, in the lean PCOS females.

## Abstract (sr)

**Uvod:**

Insulin i njegov receptor (INSR) su u vezi sa etiologijom sindroma policističnih jajnika (PCOS). U ovom radu istraživana je povezanost između polimorfizma INSR rs1799817 i PCOS kod žena iz Saudijske Arabije.

**Metode:**

Ispitivana grupa je bila sačinjena od 126 žena sa PCOS i 118 žena sa normalnom ovulacijom koje su činile kontrolnu grupu. Pribeleženi su njihovi demografski podaci i određeni su nivoi glukoze, lipida, leptina, E2, LH, FSH, T, SHBG i insulina u plazmi. Frekvencije genotipske i alelne vrednosti rs1799817 su procenjene u obe grupe, i kod žena sa PCOS i kod kontrolne grupe. Za amplifikaciju Ekon 17 INSR gena korišćena je polimerazna lančana reakcija (PCR), a amplifikovani proizvodi su analizirani direktnim sekvenciranjem. Polimorfizam jednog nukleotida (C do T) je pronađen na lokusu 10923 (His1058) od rs1799817.

**Rezultati:**

U PCOS grupi, mutantni alel T se javlja na značajno višoj frekvenciji (0,306) u poređenju sa kontrolnom grupom (0,174) (p < 0,001). On pokazuje domi nantan efekat i povećava relativni rizik od PCOS čak i kod heterozigota (RR = 2,82). Nakon stratifikacije učesnika po indeksu telesne mase, učestalost T alela bila je značajno viša kod vitkih pacijentkinja sa PCOS u poređenju sa istim pacijentkinjama iz kontrolne grupe. Gojazne pacijentkinje sa PCOS su takođe imale veću učestalost od gojaznih iz kontrolne grupe, ali razlika nije bila statistički značajna. Nekoliko parametarskih vrednosti je bilo pod uticajem INSR genotipa, posebno V/H odnosa, nivoi glukoze, lipida, insulina i insulinske rezistencije kod pacijentkinja sa PCOS.

**Zaključak:**

Polimorfizam gena INSR rs1799817 je lokus podložnosti koji je u vezi sa PCOS kod Saudijki i povezanim metaboličkim i hormonskim promenama, posebno kod vitkih žena sa PCOS.

## Introduction

Polycystic ovary syndrome (PCOS) is a highly prevalent endocrine syndrome affecting 6 to 10% of women during their reproductive age [1]. It is the most common cause of menstrual irregularity resulting from ovulatory dysfunction and hyperandrogenism. Recently, PCOS has been found to play a critical role in the development of type-2 diabetes mellitus, dyslipidemia, cardiovascular disease, endometrial carcinoma and infertility [2] [3]. Based on the diagnostic criteria of Rotterdam consensus, PCOS is diagnosed when two out of the three following criteria are met: oligomenorrhea or amenorrhea, hyperandrogenism and ultrasonographic polycystic ovarian morphology [4].

The etiology of PCOS is not apparent but can be viewed as a heterogeneous androgen excess disorder accompanied with various degrees of gonadotropic and metabolic abnormalities, determined by the interaction of multiple genetic and environmental factors. There is growing evidence, which suggests that impair ment of ovarian steroidogenesis and follicular development plays a role in PCOS [5]. Women with PCOS have broader health implications, such as dyslipidemia, including increased low-density lipoprotein (LDL) and triglyceride levels, and reduced high-density lipoprotein (HDL) [6].

Insulin resistance (or hyperinsulinemia) is a frequently encountered feature of PCOS that affects approximately 50-70% of all women with PCOS [7]. Insulin resistance features are common in PCOS but are not required for the diagnosis. Amongst the women diagnosed with PCOS and insulin resistance, 80% are obese and suffer from insulin resistance [8]. Numerous clinical studies have established an association between insulin resistance and body mass index (BMI) in PCOS patients [9] [10] [11]. Some studies, on the other hand, have shown a lack of association between insulin resistance and BMI in PCOS [12] [13]. Currently, there is no consensus about insulin resistance in lean women with PCOS [14], however, it is speculated that insulin resistance is exacerbated by obesity [11].

Different genes have been implicated in insulin action and are used as biomarkers for PCOS. *INSR* is considered as a candidate gene for PCOS susceptibility [15]. The INSR gene is located on chromosome 19 and it consists of 22 exons and 21 introns. The exons 17-21 encode the tyrosine kinase domain of the receptor, which is necessary for insulin signal transduction [16]. Mutations in these exons can cause insulin resistance and hyperinsulinemia [17] [18]. Various polymorphisms have been reported within the coding and noncoding regions of the INSR gene, in patients with PCOS [19] [20]. According to previous studies, there is a higher frequency of single nucleotide polymorphism (SNP) in exon 17 of *INSR* [21]. Particularly, a silent C/T transition at His1058 (rs1799817), in exon 17 of INSR gene has been reported to be strongly associated with PCOS in Caucasian and Han Chinese populations [12] [22] [23]. Other studies have reported no association between rs1799817 of the *INSR* gene and insulin resistance [24] [25].

Possible interactions between genetic polymorphisms, insulin resistance, and hormonal and biochemical abnormalities have not been studied in the Saudi Arabian population. Hence, we designed this present study with an aim to investigate the association between the rs1799817 in exon 17 of the *INSR* gene and PCOS and to identify correlations between mutations in this SNP and hormonal and demographic changes in different genotypes of *INSR*, in patients suffering from PCOS.

## Materials and Methods

### Study participants

This study, conducted at several private clinics, Makkah, Kingdom of Saudi Arabia, was designed as a cross-sectional, prospective, and observational study. An IRB was obtained for the study protocol from the Um al Qura University. A total of 244 women from the outpatient Gynecology Clinic were enrolled in the study, after obtaining written informed consent.

The PCOS group consisted of 126 women diagnosed according to the Rotterdam consensus (ESHRE/ASRM, 2004). The control group included 118 normo-ovulatory women who were suffering from male, tubal, or unexplained infertility. These women had normal ovulatory cycles (25-35 days) and ultrasonic ovarian morphology. They showed no endocrine abnormalities and no clinical or biochemical signs of raised androgens. The women from the control group were matched with PCOS women by age (±2 years) and body mass index (BMI±10%).

Exclusion criteria for all the subjects included congenital adrenal hyperplasia, pregnancy, hypothyroidism, hyperprolactinemia, Cushing's syndrome, anti-diabetic, anti-obesity drugs or other hormonal drugs, i.e. glucocorticoids, antiandrogens, ovulation induction agents and the use of oral contraceptives within the last six months. None of the patients showed any metabolic, neoplastic, and cardiovascular disorder or other medical illness affecting metabolism such as hepatic disorders, hepatic disorders renal disease. All participants were non-smokers.

### Anthropometric measurements

The weight and height were recorded, and BMI (weight (kg)/height in m^2^) was calculated. Individuals with a BMI>29 kg/m^2^ and <24 kg/m^2^ were considered as obese and lean, respectively. Waist and hip circumferences were measured in the standing position, to calculate the waist-hip ratio (WHR).

### Biochemical measurements

Fasting blood (5 mL) was collected from all participants between the second and fifth day of menstruation, between 08:00 to 10:00, in plain redtop tubes to measure LH, FSH, 17β-estradiol (E2), leptin, insulin and lipid levels in the serum. 2 mL blood was drawn in fluoride tubes (grey top) for glucose estimation. On 20^th^ or 21^st^ day of the menstrual cycle, 5 mL blood samples were collected to measure 17OH-progesterone (P), testosterone (T), and sex-hormone binding globulin (SHBG). The collected blood samples were immediately centrifuged, and the serum was stored at -80 °C until further analysis. The LH, FSH, E2, P, T, SHBG, leptin and insulin levels were detected via double antibody sandwich enzyme-linked immunosorbent assay (ELISA). Glucose oxidase method (Beckman Glucose Analyzer (Fullerton, CA)) was used to measure plasma glucose levels. Fasting serum triglyceride (TG), total cholesterol (TC), highdensity lipoprotein (HDL), and low-density lipoprotein (LDL) were detected using enzymatic colourimetric in vitro test on automated clinical chemistry analyzers. HOMA-IR was calculated using the formula: glucose (mmol/L x insulin (mU/L)/22.5.

### Genetic analysis

Polymerase Chain Reaction (PCR) was used to amplify Exon 17 of the *INSR* gene, and the amplified products were analyzed by direct sequencing according to the ABI Big Dye Terminator protocol on ABI 3100 Avant Genetic Analyze.

### Genotyping of exon 17

Genomic DNA was purified from peripheral blood leukocytes using commercially available Puregene Blood Kits. The fragment of interest was amplified by PCR, using primers designed via PRIMER 3 program. The primer sequences used were:

F: 5'-ACCTGCCGAACTACAACTGG-3'

R: 5'-TGAAGGAACAGGCGGTTAGT-3'

The PCR conditions used were: initial denaturation at 95 °C for 15 min, followed by 34 cycles of denaturation at 95 °C for 1 min, annealing at 55 °C for 1 min, and final extension at 72 °C for 1 min, with the final extension of 10 min at 72 °C. The PCR product was sequenced using the ABI Big Dye Terminator protocol on ABI 3100 Avant Genetic Analyzer.

### Statistical analysis

All analyses were carried out using SPSS (Statistical Package for the Social Science; SPSS Inc., Chicago, IL, USA), version 22 for MS Windows. Data for the biochemical and hormonal parameters are expressed as mean, SD, and SEM. Data in different groups were compared using Student's independent *t*-test. Categorical data were expressed as numbers and percentages and were compared using the Chisquare test (χ^2^). A p-value <0.05 was considered as statistically significant.

## Results

Demographic information along with lipids, insulin resistance, and hormonal parameters of women from PCOS and control groups and are summarized in Table 1. There was no statistically significant difference in BMI and hip circumference between the two groups, while WHR was significantly higher in the women with PCOS. Women with PCOS were at a higher risk of dyslipidemia, with significantly increased levels of cholesterol, triglycerides and LDL along with reduced levels of HDL (p=0.0001). Among the hormonal parameters, the PCOS group showed significantly higher levels of insulin, LH, E2, and testosterone accompanied with significantly lower levels of progesterone and SHBG (p < 0.05), in comparison with the control group. No difference was seen for FSH between the PCOS and the control groups.

**Table 1:** Demographic, lipid and insulin resistance parameters in PCOS patients and control group SEM: Standard error of the mean. ** Highly significant (p<0.001)

Parameters	Group	Mean	Std. Deviation	SEM	p-value
Age (years)	Control	24.576	5.558	0.511	0.002
	PCOS	26.603	4.470	0.398	
BMI (kg/m^2^)	Control	27.860	8.146	0.749	0.394
	PCOS	28.6714	6.648	0.592	
Waist (cm)	Control	81.534	18.485	1.701	0.011
	PCOS	86.960	14.375	1.280	
Hip (cm)	Control	106.470	16.454	1.514	0.341
	PCOS	104.730	11.790	1.050	
WHR	Control	0.76	0.073	0.007	0.0001**
	PCOS	0.84	0.082	0.008	
Cholesterol (mmol/L)	Control	3.636	0.561	0.051	0.0001**
	PCOS	4.329	0.860	0.076	
Triglyceride (mmol/L)	Control	0.8582	0.396	0.036	0.0001**
	PCOS	1.0875	0.426	0.037	
HDL (mmol/L)	Control	1.271	0.344	0.031	0.0001**
	PCOS	1.083	0.290	0.025	
LDL (mmol/L)	Control	1.714	0.657	0.060	0.0001**
	PCOS	2.436	0.637	0.056	
Leptin (ng/mL)	Control	26.352	20.665	1.90	0.612
	PCOS	25.183	14.974	1.334	
Fasting Insulin (pmol/L)	Control	73.647	38.557	3.549	0.0001**
	PCOS	96.887	59.408	5.295	
Fasting Glucose (mmol/L)	Control	4.72	0.492	0.045	0.0001**
	PCOS	5.04	0.517	0.046	
HOMA-IR	Control	2.2943	1.31501	.12157	0.0001**
	PCOS	3.1987	2.15287	.19179	
LH (IU/L)	Control	4.642	1.420	0.131	0.0001**
	PCOS	14.101	6.993	0.620	
FSH (IU/L)	Control	4.986	1.631	0.150	0.750
	PCOS	4.922	1.511	0.136	
LH/FSH ratio	Control	0.99	0.348	0.032	0.0001**
	PCOS	2.92	1.027	0.091	
E2 (pmol/L)	Control	135.381	69.8456	6.4298	0.0001**
	PCOS	193.355	97.0028	8.6417	
Progesterone (nmol/L)	Control	27.47	20.869	1.929	0.0001**
	PCOS	3.40	2.793	.249	
Testosterone (nmol/L)	Control	1.19	.614	.057	0.0001**
	PCOS	2.77	.763	.068	
SHBG (nmol/L)	Control	46.669	20.1307	1.8532	.0001
	PCOS	25.381	12.4905	1.1127	

Allele frequencies of rs1799817 in PCOS and control groups are summarized in Table 2. In the PCOS group, CC genotype was lower compared to the control group, while the CT genotype was significantly higher (p=0.0001). The frequency of the C allele was significantly lower while the T allele was significantly higher in PCOS patients compared to the control group (p=0.0009). The variant T allele was found to be significantly predisposing to PCOS (OR=2.02).

**Table 2:** Genotypes and allele frequencies of the INSR gene SNP, rs1799817, in patients with PCOS and the control group * significant (p<0.05). ** highly significant (p<0.001)

	Control 118 (%)	PCOS 126 (%)	OR	CI	2-value	p-value
Genotype Frequency						
CC	87 (73.7%)	64 (50.7%)	Ref			
CT	21 (17.8%)	47 (37.3%)	2.82	1.6–5.0	12.50	0.0001
TT	10 (8.5%)	15 (11.9%)	2.03	0.86–4.8	2.66	0.1
CT+TT	31 (26.3%)	62 (49.2%)	2.59	1.53–4.37	12.92	0.0003**
Allele Frequency						
C	0.826	0.694	Ref			
T	0.174	0.306	2.02	1.33–3.01	11.01	0.0009**

After the stratification of participants by BMI as shown in Table 3, we found a significantly lower frequency of the C allele in the lean PCOS patients compared to the lean control group: 31.9% of lean patients with PCOS and 11.2% of lean controls had the T allele (p=0.0001). In contrast, the frequency of the T alleles did not differ significantly between obese PCOS and lean PCOS (p=0.669). The data from the Saudi population was compared with the reports of other studies reported in the literature, and the results are presented in Table 5.

**Table 3:** Allele frequencies of the INSR gene C/T single nucleotide polymorphism in lean and obese PCOS patients and control group P1=Lean PCOS- vs. Lean Controls-; P2=Obese PCOS- vs. Obese Controls-; P3=Lean PCOS- vs. Obese PCOS-; P4=Lean Controls- vs. Obese Controls-; * significant (p<0.05). ** highly significant (p<0.001)

Genotype	Lean PCOS n=58 (%)	Lean Control n=58 (%)	Obese PCOS n=68 (%)	Obese Control n=60 (%)	*P1*	*P2*	*P3*	*P4*
CC	27 (46.6)	47 (81)	37 (64.4)	40 (66.7)	0.0006**	0.156	0.598	0.159
CT	25(43.1)	9 (15.5)	22 (32.4)	12 (20)	0.0053*	0.120	0.366	0.821
TT	6 (10.3)	2 (3.4)	9 (13.2)	6(10.0)	0.2722	1.000	0.783	0.095
CT+TT	31 (53.3)	11 (18.9)	31 (45.6)	18 (30.0)	0.0006**	0.156	0.598	0.159
Allele Frequency	Lean PCOS Chr n=116 No. (Freq)	Lean Controls n=116 No. (Freq)	Obese PCOS n=136 No. (Freq)	Obese Controls n=120 No. (Freq)	*P1*	*P2*	*P3*	*P4*
C	79 (0.681)	103 (0.888)	96 (0.706)	92 (0.793)	0.0001**	0.003*	0.669	0.048*
T	37 (0.319)	13 (0.112)	40 (0.294)	24 (0.206)				

To study the effect of this polymorphism on demographic parameters, the data in the different genotypes were separately analyzed for the insulin resistance biomarker and hormonal changes in both PCOS and control group. The results are summarized in Table 4. Within the control group, the insulin resistance, dyslipidemia and hyperandergondism parameters in the CC genotype did not differ significantly from the CT and TT genotypes. Interestingly, PCOS patients with the genotypes CC and/or CT genotype showed a significantly higher waist (p=0.028), hip (p=0.007), cholesterol (p=0.021), LDL (p=0.03) and insulin (0.047) values compared to the TT genotype. Furthermore, WHR and fasting levels of insulin and glucose differed significantly between the CT and TT genotypes. For fasting insulin, as indices of insulin resistance in PCOS, women with CC genotype showed higher insulin levels, when compared to women with CT genotype. Regarding the hormonal changes in sex steroid hormone parameters, PCOS women with CC genotype showed elevated levels of E2 (p=0.016) and progesterone (p=0.025), when compared to CT genotype.

## Discussion

There are controversial reports about the effect of obesity, indicated by BMI, waist and hip circumferences, and waist to hip ratio, on the PCOS incidence. The high values of WHR observed in this study are in agreement with a previous study done by Mutib and coworkers [26] on Iraqi PCOS women. Their study reported a significant difference in BMI, waist and hip circumferences and waist/hip ratio between PCOS women and healthy control group [27]. In this study, the waist circumferences and waist/hip ratios were higher among Saudi Arabian women with PCOS compared with those in the healthy control group. These two results support the current conclusion that there are anthropometric changes in PCOS women. The strong relationship between BMI and PCOS, combined with the known benefits of weight loss were reported in the 2010 Australian survey [9]. On the other hand, no correlation was reported between BMI and PCOS in a few other clinical and research studies [28] [29] [30]. In addition, some findings showed that women with low BMI (i.e., lean) showed a stronger association with PCOS than obese women [27] [29].

An impaired lipid profile and sex hormone parameters have been noticed in PCOS women from different populations. Regardless of BMI, women suffering from PCOS, belonging to different ethnicities, were at a higher risk of dyslipidemia (i.e., high cholesterol, TG, LDL, and low HDL), compared to their healthy counterparts [31]. Dyslipidemia is considered as a common metabolic alteration in women suffering from PCOS. Thus, many researchers have suggested treatments involving reduction of lipid levels, which in turn, have shown beneficial effects in infertile women with PCOS [32] [33]. Hyperinsulinemia and high glucose levels observed in our study confirm a substantial positive correlation between insulin resistance and PCOS in women [34] [35]. Previous research suggests that high levels of androgens seemingly affect insulin action on glucose metabolism; however, the exact mechanism is unclear [36].

Many studies have been conducted on different populations, including populations from America, Iran, China, Korea, India, Brazil, and Croatia to understand the effect of INSR SNP at exon 17 in PCOS development Table 4. This study is the first study that reports the presence of the C/T polymorphism at His1058 in exon 17 of INSR in Saudi Arabian women with PCOS; this polymorphism was strongly associated to PCOS development in Saudi Arabian women.

**Table 4:** The anthropometric, lipid and insulin resistance and sex hormone parameters in PCOS patients and control group with different genotypes of INSR H1085H (rs1799817) a=p- value for CC vs TT; b=p- value for CC vs CT; c=p- value for TT vs CT

	INSR exon 17		Control				PCOS patients		
			N		Mean	P	N	Mean	p-value
Age (years)	CC		87		24.33±0.58	0.392^a^ 0.146^b^ 0.097^c^	64	26.45±0.58	0.328^a^ 0.319^b^ 0.090^c^
	CT		21		26.33±1.22		47	27.42±0.63	
	TT		10		23.00±1.38		15	25.33±0.90	
BMI (kg/m^2^)	CC		87		27.05±0.88	0.080^a^ 0.197^b^ 0.559^c^	64	29.63±0.84	0.477^a^ 0.058^b^ 0.365^c^
	CT		21		29.63±1.75		47	27.22 ±0.98	
	TT		10		31.21±2.00		15	28.67±1.21	
Waist (cm)	CC		87		80.31±1.99	0.263^a^ 0.431^b^ 0.629^c^	64	89.98±1.83	0.335^a^ 0.012^b^ 0.171^c^
	CT		21		80.31±1.99		47	82.78±2.18	
	TT		10		87.30±5.78		15	87.13±2.27	
Hip (cm)	CC		87		104.51±1.74	0.090^a^ 0.098^b^ 0.670^c^	64	107.66±1.42	0.026^a^ 0.007^b^ 0.820^c^
	CT		21		111.10±3.53		47	101.7 ±1.74	
	TT		10		113.80±5.27		15	101.00±2.49	
WHR	CC		87		0.76±0.01	0.959^a^ 0.518^b^ 0.694^c^	64	0.85±0.01	0.162^a^ 0.042^b^ 0.005^c^
	CT		21		0.75±0.01		47	0.81±0.01	
	TT		10		0.76±0.02		15	0.88±0.02	
Cholesterol (mmol/L)	CC		87		3.65±0.06	0.899^a^ 0.530^b^ 0.794^c^	64	4.50± 0.11	0.261^a^ 0.018^b^ 0.575^c^
	CT		21		3.57±0.15		47	4.13±0.11	
	TT		10		3.63±0.19		15	4.13±0.11	
Triglyceride (mmol/L)	CC		87		0.84±0.04	0.279^a^ 0.508^b^ 0.670^c^	64	1.15±0.05	0.034^a^ 0.244^b^ 0.255^c^
	CT		21		0.90±0.10		47	1.05±0.06	
	TT		10		0.97±0.10		15	0.92±0.08	
HDL (mmol/L)	CC		87		1.27±0.04	0.705^a^ 0.899^b^ 0.696^c^	64	1.11± 0.04	0.584^a^ 0.230^b^ 0.828^c^
	CT		21		1.28±0.07		47	1.05±0.04	
	TT		10		1.23±0.12		15	1.07±0.09	
LDL (mmol/L)	CC		87		1.65±0.07	0.109^a^ 0.244^b^ 0.508^c^	64	2.55±0.08	0.559^a^ 0.014^b^ 0.273^c^
	CT		21		1.84±0.15		47	2.26±0.08	
	TT		10		2.00±0.19		15	2.45±0.15	
Leptin (ng/mL)	CC		87		24.53±2.24	0.082^a^ 0.316^b^ 0.273^c^	64	27.23±2.00	0.613^a^ 0.090^b^ 0.485^c^
	CT		21		28.78±3.53		47	22.38±2.00	
	TT		10		37.05±7.81		15	25.20±3.43	
Fasting Insulin (pmol/L)	CC		87		70.96±4.33	0.371^a^ 0.230^b^ 0.987^c^	64	100.32±7.37	0.062^a^ 0.047^b^ 0.002^c^
	CT		21		81.26 ±7.24		47	80.12±6.80	
	TT		10		81.06±10.01		15	134.58±20.44	
Fasting Glucose	CC		87		4.68±0.05	0.650^a^ 0.172^b^ 0.657^c^	64	5.10±0.07	0.025^a^ 0.754^b^ 0.009^c^
	CT		21		4.85±0.10		47	5.06±0.05	
	TT		10		4.76±0.62		15	4.75±0.12	
HOMA-IR	CC		87		2.25±0.15	0.383^a^ 0.250^b^ 0.920^c^	64	3.39±0.29	0.270^a^ 0.046^b^ 0.004^c^
	CT		21		2.57±0.26		47	2.63± 0.23	
	TT		10		2.52±0.34		15	4.12±0.64	
LH (IUL)		CC		87	4.61±0.15	0.990^a^ 0.440^b^ 0.480^c^	64	13.88±0.82	0.118^a^ 0.535^b^ 0.048^c^
		CT		21	4.60±0.32		47	13.27± 0.52	
		TT		10	5.00±0.44		15	17.64±3.57	
FSH (IUL)		CC		87	5.09±0.18	0.011^a^ 0.744^b^ 0.066^c^	64	4.84±0.16	0.367^a^ 0.753^b^ 0.406^c^
		CT		21	4.97±0.34		47	4.92±0.16	
		TT		10	4.10±0.29		15	5.28±0.55	
LH/FSH ratio		CC		87	0.96±0.04	0.011^a^ 0.803^b^ 0.048^c^	64	2.88±0.13	0.226^a^ 0.131^b^ 0.128^c^
		CT		21	0.99±0.08		47	2.84±0.12	
		TT		10	1.26±0.10		15	3.28±0.37	
E2 (pmol/L)		CC		87	130.56±7.19	0.253^a^ 0.471^b^ 0.613^c^	64	212.55±13.07	0.958^a^ 0.003^b^ 0.028^c^
		CT		21	142.94±15.0		47	160.57±11.47	
		TT		10	157.91±28.41		15	214.17±27.24	
Progesterone (nmol/L)		CC		87	28.08±2.21	0.094^a^ 0.975^b^ 0.249^c^	64	3.90±0.40	0.680^a^ 0.012^b^ 0.186^c^
		CT		21	28.01±5.35		47	2.68±0.25	
		TT		10	20.26±3.84		15	3.53±0.81	
Testosterone (nmol/L)		CC		87	1.17±0.07	0.426^a^ 0.830^b^ 0.360^c^	64	2.81±0.10	0.830^a^ 0.500^b^ 0.781^c^
		CT		21	1.15±0.11		47	2.68±0.09	
		TT		10	1.34±0.19		15	2.78±0.23	
SHBG (nmol/L)		CC		87	47.48±2.12	0.096^a^ 0.598^b^ 0.094^c^	64	24.40±1.69	0.764^a^ 0.180^b^ 0.554^c^
		CT		21	49.66±5.11		47	27.21± 1.60	
		TT		10	37.10±5.10		15	25.20±3.28	

This single C/T polymorphism has also been identified in the Indian population [22]. The H1085H (rs1799817) polymorphism in the INSR gene is a silent polymorphism, where the protein synthesized remains the same [37]. The H1085H polymorphism could play a fundamental function, as the ATP binding site responsible for phosphorus fixation during autophosphorylation responses is positioned in this region of exon 17 [38]. While it is not apparent how a synonymous polymorphism can alter the risk of insulin resistance and associated diseases such as PCOS, there is a growing body of evidence for a potential role of silent polymorphisms in altered protein function [39]. Tehrani et al. [15] have suggested that the association between *INSR* gene silent SNP (H1085H) and diseases could be due to linkage disequilibrium with nearby functional variants; as it has no potential effect on biological roles. In a previous study, no association was observed between* INSR*=rs1799817 and PCOS patients [40]. Studies have confirmed that PCOS, insulin resistance and obesity are interrelated [41] [42]. Thus, the present cross-sectional study participants were divided into lean and obese groups to investigate this interrelationship. Our comprehensive analysis found an association of this polymorphism with PCOS in lean women. CT+TT genotype was significantly higher in lean Saudi PCOS women compared to their lean control counterparts (53.3% vs 18.9%, p=0.0006). In accordance to our findings, a significantly strong association was also found between CT+TT genotype in lean Caucasian PCOS women, compared to their lean control counterparts (47 vs 29%, p=0.03) [43]. Another study in Chinese females reported a higher prevalence of CT polymorphic genotype in lean participants compared to the obese ones (52.2 vs 25.5%, p=0.01) [12]. A clinical study among Indian women with PCOS showed a higher prevalence of CT polymorphic genotype in lean women with PCOS than the obese women [22]. On the other hand, a Korean study did not reveal such associations, possibly, due to ethnic variability [13]. There was no significant difference between PCOS women with CC and CT genotypes in term of insulin and glucose levels, but CT and TT showed a strong association with both (p=0.002, 0.007 respectively).

PCOS women with CC genotype have higher levels of insulin and simultaneously, showed dyslipidemia (high cholesterol, TG, LDL, and low HDL). Insulin plays a critical role in adipose tissue through different mechanisms, including lipolysis inhibition and increased lipogenesis, TG release promotion from adipose stores, glucose transport enhancement. All these mechanisms promote dyslipidemia [44] [45].

The CC genotype was observed more frequently in both groups, i.e. PCOS cases and controls, and no significant difference was observed in anthropometric, hormonal and biochemical parameters between the genotypes. On the other hand, the frequency of the uncommon "T" allele of the INSR was significantly higher in lean patients with PCOS compared to that in the lean control group participants. These findings were consistent with the results of a previous study that investigated the T/C polymorphism in the exon region of the *INSR* gene in a Korean population [13].

The alterations in levels of testosterone (T) and Progesterone (P) in the current study were similar to findings of Baig et al. [46]. They reported higher levels of serum T and P concentrations in the PCOS women, compared to the women with normal ovulatory cycles. Numerous studies have studied SHBG at biochemical and genetic levels and shown an inverse association between insulin and SHBG levels in PCOS [47] [48]. Insulin is responsible for stimulating ovary growth and increase the action of gonadotropins on ovary steroid synthesis; via suppressing the combination of SHBG in the liver. Furthermore, PCOS women with hyperinsulinemia had low levels of SHBG. SHBG is now considered as a surrogate marker of insulin resistance in PCOS diagnosis. The results of the present study showed that women suffering from PCOS have the lowest levels of SHBG if they carry CC genotype compared with other genotypes (i.e., CT and TT) [49]. In lean PCOS, there was a significant association between CC and CT genotypes, but the influence of CT on SHBG levels was higher than CC (mean= 27.21 nmol/L, p=0.182). It was also seen that LH, E2, P, T, and A levels were significantly associated with CT and TT genotypes in PCOS without any significant differences for hormonal steroid changes.

Although there is a growing body of evidence that suggests an association between leptin and PCOS, the present study found no significant association in PCOS women compared to healthy control. Moreover, no association between different genotypes (i.e., CC, CT, and TT) in both groups -PCOS cases and control. Chakrabarti [34] has suggested that there is influence of insulin and BMI on leptin levels in females with PCOS. Insulin may mediate leptin synthesis, which in turn, would inhibit the insulinmediated promotion of gonadotropin-stimulated steroidogenesis [50]. In previous studies, leptin levels have also been associated with metabolic dysfunction, shown by an alteration in sex steroid levels repro ductive function abnormalities (in terms of decreased E2 and increased T secretions) [51].

## Conclusion

In conclusion, this research has studied the effect of *INSR* SNP at exon 17 in women with PCOS concerning hormonal and metabolic changes. The C/T polymorphism at His1058 of INSR gene was associated with PCOS development in women. This silent polymorphism is also strongly correlated with the indices of insulin resistance in the lean women with PCOS group. The hormonal changes and gene single nucleotide polymorphism of INSR may play an essential role in the occurrence of insulin resistance in patients with PCOS. The H1085H site nucleotide polymorphism of the insulin receptor gene is one of the susceptibility genes in patients with PCOS, especially in non-obese PCOS patients.

## Declarations

### Ethics approval and consent to participate

Ethical approval was obtained from the Institutional Review Board (IRB), Umm Al-Qura University, Makkah, Kingdom of Saudi Arabia (IRB No. 235) and each female was required to sign an informed consent form. All participants gave written informed consent prior to inclusion in the study.

### Consent for publication

The author gives full consent for publication of this work.

### Availability of data and material

The data is available with the author and can be provided if required. Due to ethical concerns, the data of the present study cannot be openly available.

#### Funding

This work was funded by the National Plan for Science, Technology and Innovation (MAARIFAH), King Abdul-Aziz City for Science and Technology, Kingdom of Saudi Arabia, grant Number No 08-MED604-2.

### Authors’ contributions

MD designed the study, collected and analyzed the data, and drafted and finalized the manuscript.

*Acknowledgements*. The author is grateful to the National Plan for Science, Technology and Innovation (MAARIFAH), King Abdul-Aziz City for Science and Technology, Kingdom of Saudi Arabia, grant Number No 08-MED 604-2. The author gratefully acknowledges Dr Mazin H. Daghestani, Department of Obstetrics and Gynecology, College of Medicine, Umm Al-Qura University, Makah, Kingdom of Saudi Arabia, for assistance with the recruitment of patients for the PCOS and control groups, the appreciation extends to all the subjects for their cooperation and participation in the study. The author also thanks the Deanship of Scientific Research and RSSU at King Saud University for their technical support.

### Conflict of interest statement

The authors stated that they have no conflicts of interest regarding the publication of this article.

## List of abbreviations

INSR, Insulin receptor; BMI, body massindex; E2, Eostrogen; ELISA, enzyme-linked immunosorbentassay; FSH, Follicular stimulating hormone; HDL, high-densitylipoprotein; IRB, Institutional Review Board; LH, Lutenizing hormone; LDL, low-density lipoprotein; P, 17OH-progesterone (P;)PCOS, polycystic ovarian syndrome; PCR, Polymerase chainreaction; SEM, Standard Error on the Mean; SHBG, sex-hormone binding globulin; SNP, single nucleotide polymorphism; SPSS, Statistical Package for the Social Science; T, Testosterone; TG, triglyceride; TC, total cholesterol (TC); WHR, W/H, waisthip ratio; c, Chi-square test.

**Table 5:** Genotypic and allelic distribution of *INSR* H1085H (rs 1799817) in PCOS patients and control group in differentpopulations

Population	Control			PCOS			p-value
	No.	Genotypes No. (%)	Alleles (frequency)	No.	Genotypes No. (%)	Alleles (frequency)	
		CC/CT/TT	C/T		CC/CT/TT	C/T	
British [52]	8	4/4/0 (50/50/0)	12/4(75/25)	22	11/11/0 (50/50/0)	33/11(78/25	0.641
American [43]	136	93/43 (68.4/31.6)	ND	99	62/37 (62.6/37.4)	ND	ND
Chinese [14]	40	35/5 (87.5/12.5)	ND	120	71/49(59.2/40.8)	ND	ND
Korean [53]	100	46/40/14 (46/40/14)	132/68 (0.66/0.34)	132	63/59/10 (47.7/44.7/7.6)	185/79 ( 0.7/0.3)	0.554
Indian [22]	144	76/56/12 (52.8/38.9/8.3)	208/80 (0.72/0.28)	180	79/77/24 (43.9/42.8/13.3)	235/125 (0.65/0.35)	0.934
Brazilian [54]	64	38/20/6 (59.4/31.3/9.4)	96/32 (0.75/0.25)	65	40/22/3 (61.5/33.8/4.6)	102/280 (78/0.22)	0.411
Croatian [55]	175	115/53/7 (66/30.3/4.0)	283/67 (0.81/0.19)	150	95/51/4 (63.3/34.0/2.7)	241/59 (0.80/0.20)	0.960
Iranian [15]	156	7/54/95 (4.5/34.6/60.9)	68/244 (0.22/0.78)	186	15/57/114 (8.1/30.6/61.3)	87/285 (0.23/0.77)	0.982
Japanese [56]	99	35/43/21 (35.4/43.4/21.2)	113/85 (0.57/0.43)	61	29/21/11 (47.5/34.4/18.0)	79/43 (0.65/0.35)	0.528
Turkish [25]	50	0/29/21 (0/58/42)	29/71 (0.29/0.71)	44	0/22/22 (0/50/50)	22/66 (0.25/0.75)	0.015
Indian [57]	50	4/20/26 (8/40/52)	28/72 (0.28/0.72)	50	17/22/11 (34/44/22)	56/44 (0.56/0.44)	0.001
